# Aging and Discoloration of Red Lead (Pb_3_O_4_) Caused by Reactive Oxygen Species Under Alkaline Conditions

**DOI:** 10.3390/molecules30102136

**Published:** 2025-05-12

**Authors:** Zhehan Zhang, Qin Huang, Jiaxing Sun, Qilong Hao, Wenyuan Zhang, Zongren Yu, Bomin Su, Haixia Zhang

**Affiliations:** 1 State Key Laboratory of Applied Organic Chemistry, College of Chemistry and Chemical Engineering, Lanzhou University, Lanzhou 730000, China; zhangzhh2024@lzu.edu.cn (Z.Z.); hqin2023@lzu.edu.cn (Q.H.); sunjx2021@lzu.edu.cn (J.S.); haoql2023@lzu.edu.cn (Q.H.); 2 Gansu Provincial Research Center for Conservation of Dunhuang Cultural Heritage, Dunhuang Academy, Dunhuang 736200, China; zhangwy@dha.ac.cn (W.Z.); yuzr@dha.ac.cn (Z.Y.); subm@dha.ac.cn (B.S.)

**Keywords:** red lead, reactive oxygen species, alkaline condition, discoloration

## Abstract

Red lead (Pb_3_O_4_) has been extensively utilized as a red pigment for centuries. However, the discoloration and blackening of red lead in historical paintings have significantly compromised the aesthetic value of mural artworks. Investigating the mechanisms behind the blackening of Pb_3_O_4_ is of paramount importance. This study examined the effects of four kinds of reactive oxygen species (ROS) on the aging process of Pb_3_O_4_ in an alkaline environment. Specifically, singlet oxygen (^1^O_2_), superoxide radical (O_2_^−^·), hydrogen peroxide (H_2_O_2_), or peroxynitrite (ONOO^−^) was individually reacted with Pb_3_O_4_. The resulting products were analyzed qualitatively and quantitatively using X-ray diffraction (XRD), X-ray photoelectron spectroscopy (XPS), scanning electron microscopy–energy-dispersive spectroscopy (SEM-EDS), Raman spectroscopy, inductively coupled plasma mass spectrometry (ICP-MS), and UV-Vis spectroscopy. The findings indicate that singlet oxygen (^1^O_2_) and superoxide radicals (O_2_^−^·) effectively induce the aging of Pb_3_O_4_, whereas hydrogen peroxide (H_2_O_2_) and peroxynitrite (ONOO^−^) exhibit little impact on its aging. This research elucidates the aging mechanisms of Pb_3_O_4_ in alkaline environments and provides valuable insights for the preservation and restoration of mural paintings.

## 1. Introduction

Red lead (Pb_3_O_4_), owing to its vibrant red color, was extensively utilized as both a cosmetic and pigment in ancient times [1,2]. Over time, red lead pigment ages and changes from red to black, significantly affecting the aesthetics of wall paintings [3]. In order to mitigate the detrimental impacts of aging-induced darkening of red lead pigment on the artistic and aesthetic integrity of murals, it is important to study the causes and mechanisms of aging and blackening of red lead. Generally, it is believed that Pb_3_O_4_ turns black due to PbO_2_ generation [4]. The lead element (Pb) of Pb_3_O_4_ has two valence states, Pb (II) and Pb (IV), so Pb_3_O_4_ can be written as 2PbO·PbO_2_ [5]. Pb (II) can be dissolved in the general acidic environment, whereas Pb (IV) remains insoluble. Previous investigations have proposed two principal mechanisms for Pb_3_O_4_ degradation. The first is the acid-induced transformation hypothesis, which suggests that Pb_3_O_4_ undergoes conversion to PbO_2_ in acidic environments. Y. Zhao et al. conducted comprehensive characterization of naturally and artificially aged Pb_3_O_4_ pigments and postulated that the chromatic alteration mechanism in Song Dynasty murals primarily involves interactions with hydrogen ions [6]. The other hypothesis is that ultraviolet (UV) light interacts with the Pb_3_O_4_ pigments. Zhao et al. transformed trace Pb_3_O_4_ pigments into pure black flaky PbO_2_ after 60 days of aging by UV light at an intensity of 600 W/m^2^ and wavelength of 313 nm [7], and Athanassiou et al. also suggested that the color of the frescoes would change under the action of UV light [8]. Previous studies have characterised the aging products by a variety of methods, such as XRD [9,10,11], XPS [12], SEM-EDS [12,13], ICP-MS [14], FT-IR [15,16], Raman spectroscopy [17,18,19] and UV-Vis [20,21]. Prior studies were typically confined to one or two characterization techniques for validating red lead aging outcomes. Given that Pb_3_O_4_ aging constitutes a time-dependent chemical process, it is not easy to understand the mechanism by reliance on single analytical methodology. It is therefore important to adopt multi-dimensional characterization techniques for synergistic analysis, which effectively eliminates systematic bias and ensures data reliability.

However, both existing hypotheses have their own limitations. The theory of acid corrosion can explain the aging of mural paintings in humid or corrosive areas, but in areas such as the Mogao Grottoes, where the mural substrate is made of alkaline gypsum, the wall environment is weakly alkaline. Acid corrosion theory does not explain the discoloration of lead paint in this environment. The ultraviolet light theory believes that the strong ultraviolet environment makes the valence electrons of Pb atoms obtain energy and be oxidized, but some murals are painted in caves, where the ultraviolet light is weak, unable to provide enough energy to activate the valence electrons of Pb, so it cannot explain the discoloration of lead pigment in this environment. Therefore, in areas with sunlight and alkaline environment at the same time (such as the Mogao Grottoes), the discoloration mechanism of red lead pigment on the murals is still unclear.

Reactive oxygen species (ROS) are strongly reactive substances, produced by oxygen in the process of electron transfer, which play an important role in the decomposition of substances in the environment [22]. ROS include mono-oxygen (^1^O_2_), hydrogen peroxide (H_2_O_2_), superoxide radical (O_2_^−^·), peroxynitrite (ONOO^−^), sulfate radical (SO_4_^−^·), etc. They are easily produced in environments with ultraviolet radiation, high oxygen concentrations, and redox conditions [23,24,25]. In addition, there is also a certain amount of reactive oxygen species in the air with a relatively mild environment, which is caused by the interaction between ozone and nitrogen oxides and the components in the air [26,27].

Since reactive oxygen species exist widely in the air and are highly oxidizing, we believe that it is possible to oxidize lead pigment and make its color black if ROS in the air collide with the lead pigment. In this investigation, the effects of ROS on the aging and darkening of Pb_3_O_4_ pigments were studied. The above four different ROS were used to age Pb_3_O_4_ under alkaline conditions, and the samples obtained at different aging times were qualitatively and quantitatively analyzed by multiple analytical techniques, such as XRD, XPS, SEM-EDS, ICP-MS, Raman spectroscopy, and UV-Vis. The purpose of this paper is to explain the discoloration of Pb_3_O_4_ pigment in an alkaline environment and provide a new idea for the restoration of cultural relics in an alkaline environment.

## 2. Materials and Methods

We first studied the aging of Pb_3_O_4_ by O_2_^−^· under alkaline conditions and analyzed the products between O_2_^−^· and Pb_3_O_4_ at different times in detail. After that, we expanded the experiments to three other types of ROS and compared the extents of aging of Pb_3_O_4_ under same conditions.

### 2.1. Preparation of Four Kinds of ROS

Superoxide free radical (O_2_^−^·) was prepared by the reaction of pyrogallol with oxygen under alkaline conditions. Singlet oxygen (^1^O_2_) is prepared by adding 30% H_2_O_2_ to NaClO (aq). Hydrogen peroxide (H_2_O_2_) is prepared directly with 30% H_2_O_2_. Peroxynitrite (ONOO^−^) is prepared by adding 30% H_2_O_2_ to NaNO_2_ (aq). The reactions are shown as (1)–(3):

(1) $$C_{6}H_{3}{\left(\mathrm{OH}\right)}_{3}+O_{2\;}\overset{{\mathrm{OH}}^{-}}{\to }\;\;C_{6}H_{3}O{\left(\mathrm{OH}\right)}_{2}^{-}\cdot +{\;H}^{+}+{\;O}_{2}^{-}\cdot$$

(2) ClO−+H2O2→Cl−+H2O+O 12

(3) $${NO}_{2}^{-}+H_{2}O_{2}\;\to \;H_{2}O+{\mathrm{ONOO}}^{-}$$

### 2.2. Aging of Pb_3_O_4_ Using Superoxide Radical Under Alkaline Conditions

**Scheme 1:**
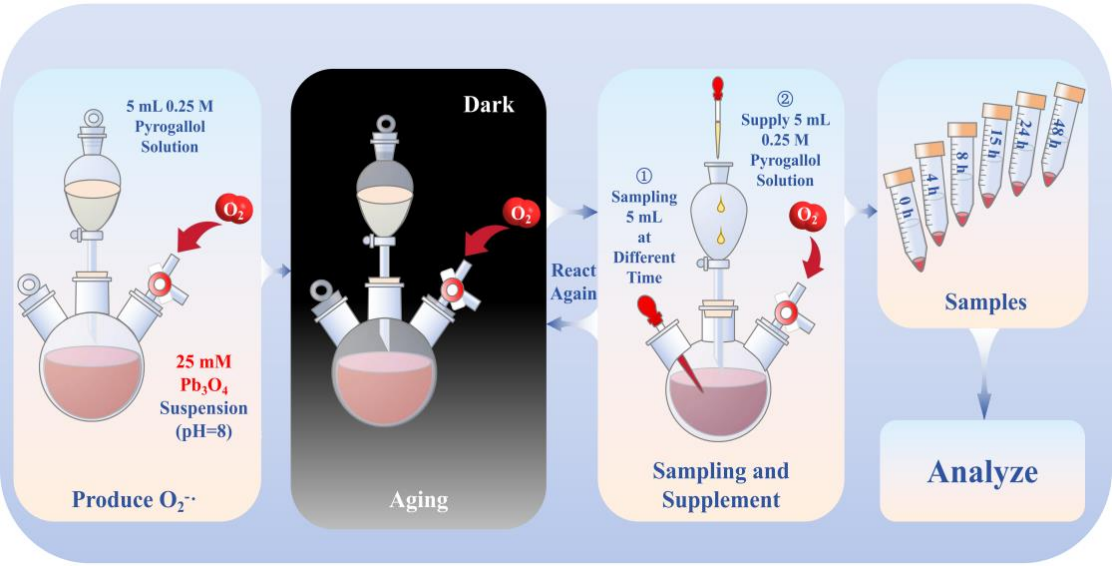
The process of aging of Pb_3_O_4_ using superoxide radical under alkaline conditions.

Pb_3_O_4_ (1.25 mmol) was combined with a NaOH solution (50 mL, pH 8.0) and subjected to ultrasonication for 30 min to prepare a 25 mM suspension. The dropping funnel contained 5 mL 0.25 M (1.25 mmol) pyrogallol solution, which was added slowly the Pb_3_O_4_ suspension in specified time intervals (0 to 4 h, 4 to 8 h, 8 to 15 h, 15 to 24 h, and 24 to 48 h). Aliquots (5 mL) were collected at specified time intervals (0, 4, 8, 15, 24, and 48 h). With the exception of the final time point, each sample was immediately supplemented with 5 mL 0.25 M pyrogallol solution via the dropping funnel to maintain superoxide radical concentrations. The reaction system was maintained under a controlled oxygen atmosphere throughout the experiment. Molecular oxygen was generated through catalytic decomposition of 30% H_2_O_2_ using MnO_2_, with gas delivery regulated through a three-way valve into the whole reaction system. The detailed experimental procedure is shown in Scheme 1.

### 2.3. Aging Effects of Three Other Different ROS on Pb_3_O_4_

The other ROS-generating systems (H_2_O_2_, ^1^O_2_, and ONOO⁻) were prepared and each reacted with 1.25 mmol portions of Pb_3_O_4_ at identical concentrations for 72 h. The reaction conditions and sampling are the same as those in part 2.2.

### 2.4. Analytical Techniques

The color change of the aged sample was quantified using a recording colorimeter (LS171, Linshang, Shenzhen, China). The measurements were conducted using a D65 standard illuminant with an 8 mm measuring aperture. Thirty successive measurements were performed on pure Pb_3_O_4_, pure PbO_2_, and superoxide radical-aged samples, with a 3-s interval between each measurement. The obtained data were averaged to determine the tristimulus coordinates. The chromaticity coordinates of samples were subsequently converted to CIE 1976 L*a*b* color space through the chromameter’s proprietary software.

The electron paramagnetic resonance (EPR) test was conducted using an electron paramagnetic resonance spectrometer (ER200DSRC10/12, Bruker, Billerica, MA, USA), which operates in the X-band (9.5 GHz), with a microwave power of 2 mW and a modulation frequency of 100 kHz. All measurements were carried out using quartz capillary sample holders.

The X-ray diffraction (XRD) patterns were recorded on an Ultima IV diffractometer (Rigaku, Tokyo, Japan) using CuKα radiation (λ = 1.5418 Å) at 40 kV and 40 mA. Data collection spanned 10–60° (2θ) with a step size of 0.02° and scanning rate of 4°/min using a scintillation counter detector.

The scanning electron microscope–energy-dispersive spectrometer (SEM-EDS) images were determined on an Apreo 2S scanning electron (ThermoFisher, Waltham, MA, USA). First, the samples were adhered to the sample stage using conductive adhesive. Then, the samples were tested under a high vacuum degree (10^−6^ Pa) with an accelerating voltage of 30.0 kV and an accelerating current of 0.8 nA. Image collection was carried out using the secondary electronic mode. Finally, EDS analysis was performed using the software Bruker Esprit 2.5.

X-ray photoelectron spectroscopy (XPS) was used to analyze the surface elemental composition by using Al Kα radiation (Axis Supra XPS system, Shimadzu, Kyoto, Japan).

Lead element quantification was performed on an inductively coupled plasma mass spectrometer (ICP-MS), Plasma Quant PQ9000 (Analytik Jena AG, Jena, Germany).

Raman spectroscopy was performed using a high-resolution ultra-sensitive intelligent Raman imager (HORIBA, Kyoto, Japan) with a 532 nm He-Ne laser as an excitation source.

UV-Vis spectra were scanned by ultraviolet-visible spectrophotometer (T700, Persee, Beijing, China), using tungsten filament as the light source to scan the visible light absorption spectrum at 500–800 nm. Finally, the obtained data were processed on Origin 2021.

The calculation methods and conditions used are as follows. All electronic calculations were performed with the Gaussian 16 package. The quantum chemical calculations were performed with the Gaussian 16 package, revision D.01, in addition to GaussView 6.0, which was used for visualization of the optimum structures, molecular frontier orbitals for molecules in reactions. The set temperature was 298.15K, the pressure was 1 atm, the solvent model was IEFPCM, and the solvent was water. Density functional theory (DFT) was used to optimize the ground state geometry of molecules in reactions at the M06-2x/def2-TZVPP level and D3 correction. The molecular configuration was obtained by ensuring no virtual frequency through frequency calculation and vibration analysis calculation. The energy was calculated using the M06-2X functional in conjunction with the def2-TZVPP basis set and D3 correction. Finally, the thermodynamic data were calculated using Shermo 2.6 software to obtain the results.

## 3. Results and Discussion

### 3.1. Confirmation of the ROS Production

**Figure 1:**
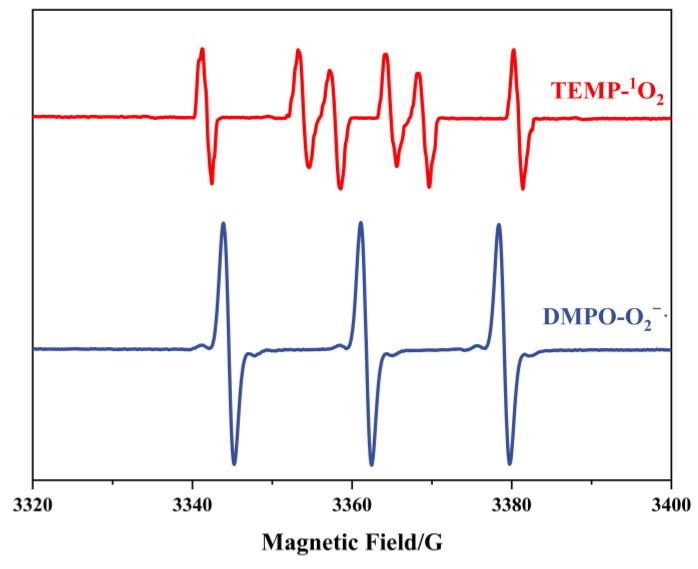
EPR spectra of TEMP- ^1^O_2_ and DMPO-O_2_^−^·.

To confirm the generation of radicals, EPR analysis was employed. Given that both H_2_O_2_ and ONOO^−^ exist as stable molecular/ionic species under the preparation conditions, we focused exclusively on detecting superoxide radicals (O_2_^−^·) and singlet oxygen (^1^O_2_). The detection protocol involved using DMPO (5,5-dimethyl-1-pyrroline N-oxide) and TEMP (2,2,6,6-tetramethylpiperidine) as spin-trapping agents for O_2_^−^· and ^1^O_2_, respectively. Subsequent EPR characterization of the trapped adducts revealed distinct spectral signatures. As illustrated in Figure 1, the characteristic peaks corresponding to DMPO-O_2_^−^· (g = 2.006, aN = 14.2 G, aH = 11.3 G) and TEMP-^1^O_2_ (triplet signal with aN = 16.5 G) exhibited remarkable intensity in the EPR spectra. These observations provide conclusive evidence that the described preparation methodology effectively generates superoxide radicals or singlet oxygen species.

### 3.2. Chromaticity Coordinates

**Figure 2:**
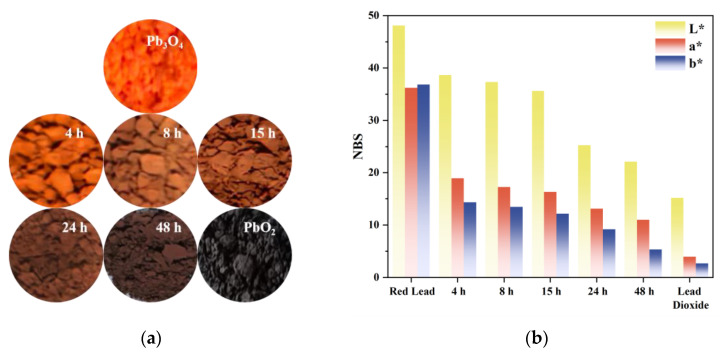
(**a**) Photos of pure Pb_3_O_4_, five aged samples and pure PbO_2_; (**b**) Variation trend of chrominance coordinate parameters.

Visual observation revealed that the superoxide radical-reacted specimens exhibited progressive chromatic transition from the characteristic red hue of pristine Pb_3_O_4_ to a PbO_2_-like black appearance (Figure 2a). The variation trend of L*a*b* chromaticity parameters of the sample is shown in Figure 2b. The luminance parameter L* demonstrated a value range of 48.11–15.19, while the a* coordinate (representing the red-green axis) varied between 36.24 and 3.97, and the b* coordinate (indicating the yellow-blue axis) decreased from 36.85 to 2.69. This acquired veridical colorimetric data establish a quantitative foundation for investigating the chromogenic alteration mechanism during the discoloration process.

### 3.3. Crystal Structure

**Figure 3:**
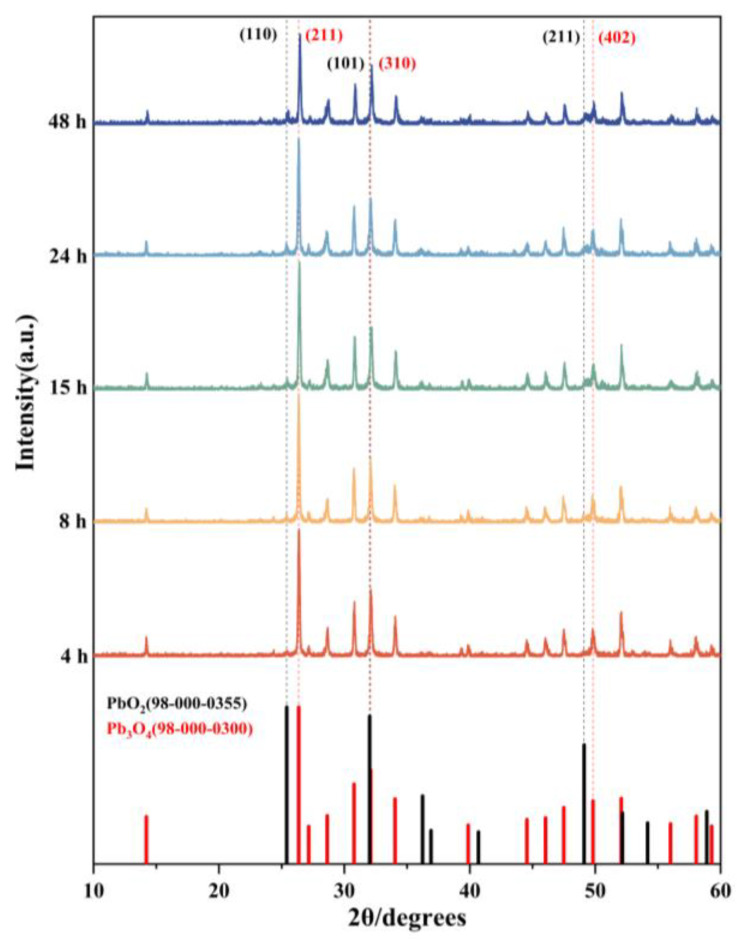
XRD patterns of products aged of Pb_3_O_4_ with O_2_^−^· at different times and standard PDF of Pb_3_O_4_ and PbO_2_.

XRD analysis was performed on reaction products obtained at different time intervals during the reaction. The results revealed that the aging product of lead tetroxide (Pb_3_O_4_) was lead dioxide (PbO_2_). The XRD diffraction patterns of respective phases are presented in Figure 3. Characteristic peaks for Pb_3_O_4_ were identified at 26.36° (211), 32.10° (310), and 49.81° (402), while those corresponding to PbO_2_ appeared at 25.40° (110), 32.01° (101), and 49.11° (211), demonstrating close proximity in peak positions between the two phases. Three key observations emerge from the diffraction pattern evolution: first, in the 25–27° range, the initially smooth profile at 26.36° developed into a distinct sharp peak with increasing reaction time; second, in the 32–33° region, since the characteristic peaks of Pb_3_O_4_ and PbO_2_ overlap, we used the ratio of the target peak to its left neighbor to determine whether a phase transition existed. It can be seen from the figure that the intensity ratio of the adjacent peaks to its left increases gradually, so it can be judged that PbO_2_ was generated; third, the 49–50° range showed gradual emergence of a defined peak at 49.11° over time. These systematic changes collectively indicate phase transformation dynamics: the diminishing Pb_3_O_4_ peak intensities accompanied by growing PbO_2_ signatures confirmed partial oxidation of pristine Pb_3_O_4_ to PbO_2_. The crystalline structure evolution suggests gradual replacement of the original Pb_3_O_4_ matrix with PbO_2_ domains, resulting in the products comprising a composite of both phases.

### 3.4. XPS Analysis

**Figure 4:**
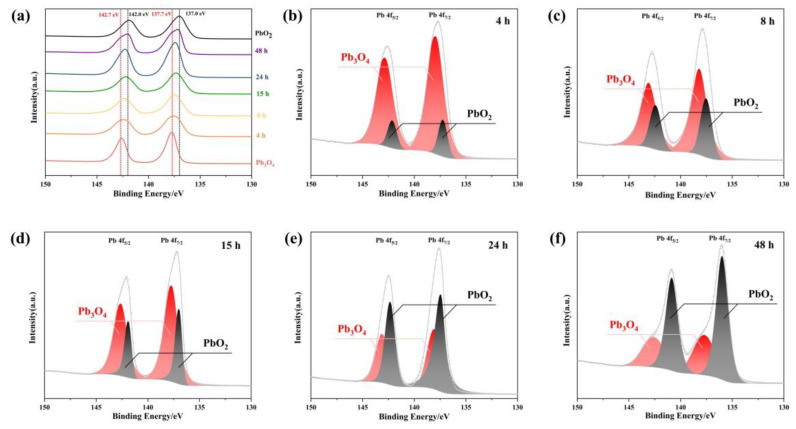
(**a**) The XPS peak shift of the product of Pb_3_O_4_ with O_2_^−^· at different reaction times; (**b**–**f**) Pb peak division of reaction products of different times: (**b**) 4 h; (**c**) 8 h; (**d**)15 h; (**e**) 24 h; (**f**) 48 h.

To verify whether the reaction product of Pb_3_O_4_ with O_2_^−^· is PbO_2_, XPS analysis was conducted to determine the oxidation states of Pb species. As shown in Figure 4, the Pb 4f spectra observed in the 130–150 eV range can be deconvoluted into two characteristic spin-orbit components corresponding to Pb 4f 5/2 and Pb 4f 7/2. Reference data indicate that Pb_3_O_4_ exhibits characteristic binding energies of 137.7 eV (4f 7/2) and 142.7 eV (4f 5/2), while PbO_2_ demonstrates slightly lower binding energies at 137.0 eV (4f 7/2) and 142.0 eV (4f 5/2), as marked by black and red dashed lines in Figure 4a, respectively [28]. The temporal evolution of Pb 4f spectra in Figure 4a reveals a progressive shift of both spectral peaks from the characteristic positions of Pb_3_O_4_ (137.7 eV and 142.7 eV) toward those of PbO_2_ (137.0 eV and 142.0 eV) with increasing reaction duration with superoxide radicals. This energy shift provides direct evidence of the chemical transformation from Pb_3_O_4_ to PbO_2_ during the radical-mediated oxidation process. To quantitatively analyze this conversion, peak deconvolution was performed on the Pb 4f spectra at different reaction intervals. The spectral components were assigned to either Pb_3_O_4_ (137.7/142.7 eV) or PbO_2_ (137.0/142.0 eV) through Gaussian–Lorentzian curve fitting, as presented in Figure 4b–f. The progressive enhancement of PbO_2_-associated peak intensities (137.0 eV and 142.0 eV) relative to the diminishing Pb_3_O_4_ components demonstrates a time-dependent increase in PbO_2_ formation. This observation aligns with the hypothesis that superoxide radicals facilitate the oxidation of Pb (II) in Pb_3_O_4_ to Pb (IV) in PbO_2_ through electron transfer processes. The quantitative correlation between reaction duration and PbO_2_/Pb_3_O_4_ ratio, as revealed by peak area analysis, further confirms the gradual conversion mechanism. The systematic transition in Pb oxidation states provides spectroscopic evidence for the formation of PbO_2_ as the primary product in the superoxide-mediated oxidation of Pb_3_O_4_.

### 3.5. Raman Spectroscopy

**Figure 5:**
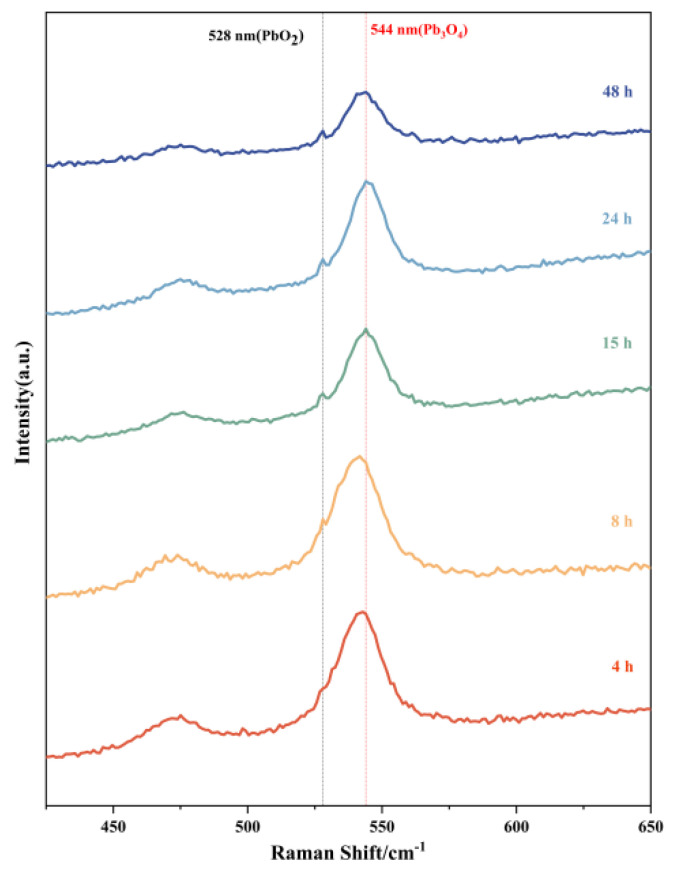
Raman spectroscopy of reaction products of Pb_3_O_4_ with O_2_^−^· at different reaction times.

Raman spectroscopic analysis was systematically performed on products obtained at different reaction intervals, as presented in Figure 5. The molecular fingerprint signatures of Pb_3_O_4_ and PbO_2_ exhibit distinct differences in the Raman spectral patterns. Previous literature indicates that the characteristic Raman peak of PbO_2_ typically appears at 528 nm, while Pb_3_O_4_ demonstrates its primary vibrational mode at 544 nm [18,29,30,31]. These reference positions are marked in the figure with black and red dashed lines, respectively. The spectral evolution reveals significant insights into the reaction progression. All five time-dependent products consistently display prominent peaks at 544 nm, confirming the persistent presence of unreacted Pb_3_O_4_ throughout the experimental duration. Notably, the characteristic PbO_2_ signal at 528 nm underwent progressive intensification with extended reaction time, manifested through both peak sharpening and amplitude enhancement. For the 4 h product, while no distinct PbO_2_ peak is observable at 528 nm, careful examination reveals a pronounced shoulder peak in this spectral region. This spectral feature can be attributed to the vibrational coupling between residual Pb_3_O_4_ and nascent PbO_2_ phases, suggesting initial stage formation of the latter compound. The temporal evolution of these spectral characteristics provides compelling evidence of the gradual chemical transformation from Pb_3_O_4_ to PbO_2_ with prolonged reaction duration. This experimental observation aligns well with the proposed reaction mechanism that Pb_3_O_4_ serves as the precursor material undergoing oxidative conversion to PbO_2_.

### 3.6. Ratio of Lead and Oxygen Elements in Aging Products

Systematic elemental characterization was performed on reaction products obtained at different durations to investigate the inorganic transformation mechanism. Based on stoichiometric calculations, Pb_3_O_4_ exhibits a Pb:O atomic ratio of 3:4, whereas PbO_2_ demonstrates a reduced ratio of 1:2. These ratios indicate a progressive decrease in both atomic and mass ratios of Pb to O during the conversion from Pb_3_O_4_ to PbO_2_. Furthermore, the mixed oxidation states of Pb in Pb_3_O_4_ comprise Pb (II) and Pb (IV) in a 2:1 ratio, while PbO_2_ exclusively contains Pb (IV) species. This distinct oxidation state distribution implies a systematic decline in the Pb (II)/Pb (IV) ratio during the transformation process.

Two analytical approaches were employed: EDS for elemental mass and atomic ratio and ICP-MS with redox speciation analysis to monitor Pb (II)/Pb (IV) evolution. Since the prepared sample for EDS was thin enough, it can be regarded that lead and oxygen atoms had covered the surface of the conductive adhesive. Therefore, through EDS image analysis, the percentages of lead atoms and oxygen atoms on the sample surface to the total number of atoms could be obtained.

The method for determining the Pb (II)/Pb (IV) ratio in a sample by ICP-MS is as follows. Specifically, 0.5 g of reaction product was dispersed in water and stirred for 2 h to ensure complete dissolution, followed by phase separation through centrifugation. The supernatant with a certain volume was collected to ICP-MS analysis for Pb (II) content (denoted as n_1_). The residual precipitate was subsequently treated with 2 M HNO_3_ solution with 2 h of stirring for complete dissolution, followed by secondary centrifugation. The resulting supernatant was similarly volume quantified and analyzed by ICP-MS to determine additional Pb (II) content (n_2_). The remaining insoluble precipitate, identified as PbO_2_, was dried and weighed to determine Pb (IV) content (n_3_). The Pb (II)/Pb (IV) ratio was calculated using the following equation:

(4) $$\frac{\mathrm{Pb}\left(\mathrm{II}\right)}{\mathrm{Pb}\left(\mathrm{IV}\right)}=\frac{n_{1\;}+{\;n}_{2}}{n_{3}}$$

**Figure 6:**
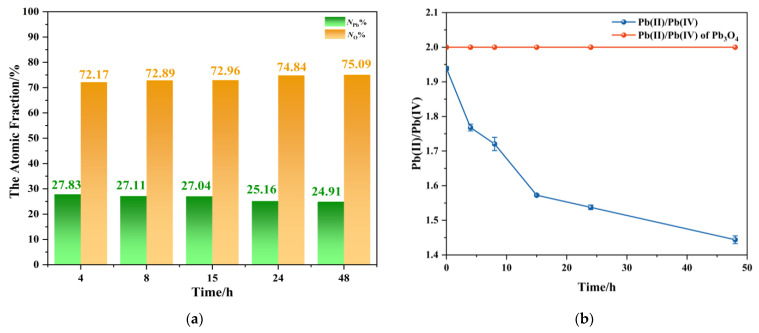
(**a**) The atomic fraction of lead/oxygen atoms in the total number of atoms of the product between Pb_3_O_4_ with O_2_^−^· at different reaction times; (**b**) the ratio of Pb (II) to Pb (IV) in reaction products at different times measured by ICP-MS.

The analysis results from products with different aging times obtained by using the above two methods are shown in Figure 6. EDS elemental analysis proved that there were only Pb and O elements in these products, and the atomic fractions of Pb and O are shown in Figure 6a. The EDS images of the products at different reaction times are shown in Figure S1. The results showed that with the extension of the reaction time, the percentage of lead atoms in the total number of atoms systematically decreased, and in contrast to oxygen atoms. These trends are consistent with the stoichiometric transformation from Pb_3_O_4_ to PbO_2_, related to the oxidation of Pb_3_O_4_. Figure 6b presents the temporal profile of Pb (II)/Pb (IV) molar ratios calculated, where the red dashed line represents the theoretical stoichiometric ratio (2:1) for pristine Pb_3_O_4_, and the blue curve corresponds to experimentally determined Pb (II)/Pb (IV) molar ratios in the products. It can be found that a monotonic decrease in Pb (II)/Pb (IV) ratio with reaction duration reflected the progressive oxidation of Pb (II) to Pb (IV). It is worth noting that the ratio of Pb (II)/Pb (IV) of the blue line is less than 2 at the beginning, which is due to the inevitable loss of Pb^2+^ during ICP-MS pre-treatment. Despite this systematic underestimation, the pronounced ratio reduction (the ratio at 48 h is about 1.4) overwhelmingly confirmed that oxidation took place. The decrease in Pb/O ratio associated with oxygen accumulation and the decrease in Pb (II)/Pb (IV) values provide strong evidence of the transformation pathway proposed in this research.

In addition, we also used the oxidation discoloration system of TMB at pH = 3 [31] to prove that PbO_2_ increases and Pb_3_O_4_ decreases in aging products with the increase of aging time, which is shown in the Supporting Information.

### 3.7. Comparison of Aging Effects of Different ROS

Following confirmation of superoxide radicals’ oxidative impact on Pb_3_O_4_, we systematically evaluated the oxidation efficiencies of different ROS toward Pb_3_O_4_ with the same reaction conditions and prolonged the reaction time to 72 h.

**Figure 7:**
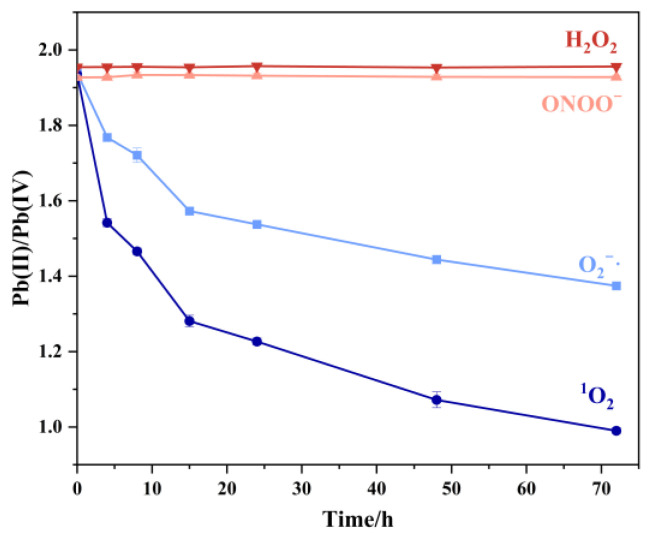
Pb (II)/Pb (IV) in aging products of Pb_3_O_4_ treated with four free radicals at different reaction times under the conditions shown in part 2.2.

As shown in Figure 7, hydrogen peroxide demonstrated limited oxidation capacity, attributable to the nanozyme-like catalytic activity of submicron Pb_3_O_4_ particles that accelerated H_2_O_2_ decomposition, thereby depleting available oxidizing equivalents. Peroxynitrite exhibited low oxidation efficiency too, and ^1^O_2_ was the most effective ROS for Pb_3_O_4_ oxidation among the four kinds of ROS. The experiment conducted using the TMB-UV-Vis system, as shown in Figure S4, also well confirmed the oxidation of Pb_3_O_4_ by ^1^O_2_.

### 3.8. Gibbs Free Energy Calculation

Pb_3_O_4_ is expected to react with the ROS as shown by reactions (5)–(8) to form the oxidation product PbO_2_.

(5) Pb3O4+O 12 → 3PbO2

(6) $$3{\mathrm{Pb}}_{3}O_{4\;}+4O_{2}^{-}\cdot +2H_{2}O\;\to \;9\mathrm{Pb}O_{2}+4{\mathrm{OH}}^{-}$$

(7) $${\mathrm{Pb}}_{3}O_{4}+2{\mathrm{ONOO}}^{-}\;\to \;3\mathrm{Pb}O_{2}+2{\mathrm{NO}}_{2}^{-}$$

(8) $${\mathrm{Pb}}_{3}O_{4}+{2H}_{2}O_{2}\;\to \;{3\mathrm{PbO}}_{2\;}+H_{2}O$$

**Figure 8:**
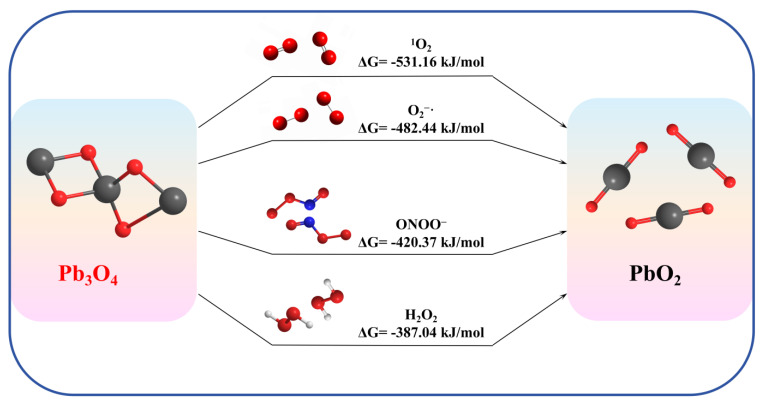
Gibbs free energy change calculation for four ROS reactions.

To explain the observed faster reaction kinetics between Pb_3_O_4_ and different ROS, we performed Gibbs free energy change (ΔG) calculations for reactions (5)–(8), with results summarized in Figure 8. All thermodynamic parameters for determining ΔG are shown in Table S1.

Thermodynamic analysis reveals that all investigated reactions, reactions (5) to (8), exhibit negative ΔG values (ΔG < 0), confirming their thermodynamic spontaneity under experimental conditions. Notably, the calculated ΔG(^1^O_2_) is a greater negative value than the ΔG(O_2_^−^·), providing a robust thermodynamic basis for the enhanced aging efficiency of ^1^O_2_ over O_2_^−^·. Intriguingly, while H_2_O_2_ and ONOO^−^ also demonstrate thermodynamic feasibility for Pb_3_O_4_ oxidation, their experimental validation proved less conclusive in this study. It may be due to the nano-enzyme nature of Pb_3_O_4_ and the instability of ONOO^−^ in this environment, making the corresponding reaction difficult to happen.

### 3.9. Discussion

We discovered that certain ROS can oxidize Pb_3_O_4_ into black PbO_2_, causing the darkening of red pigments in murals and consequent loss of aesthetic value. These ROS in natural environments may originate from UV-irradiated atmospheric components, soil matrices, aqueous systems, or microbial lysis processes [32,33,34,35,36]. The proposed discoloration mechanism can be elucidated as follows: In typical mural preservation environments, ultraviolet radiation-induced atmospheric reactions and moisture volatilization from wall surfaces coexist with microbial activity and decomposition in adjacent soil substrates. Moreover, the traditional red lead pigments involved pyrometallurgical processing of lead, sulfur, and nitrate precursors. Constrained by the technological parameters of ancient time, the resultant Pb_3_O_4_ pigments inherently contained intrinsic crystallographic defects including oxygen vacancies (OVs) and lead vacancies (PbVs). These structural imperfections create localized high-energy sites within the crystalline lattice that facilitate oxygen activation upon atmospheric exposure. Specifically, molecular oxygen (O_2_) adsorbed at these vacancy sites undergoes electronic state modifications, lowering the activation energy barrier for radical generation. This defect-mediated process ultimately enables the production of oxidative radicals capable of initiating and propagating the degradation of red lead pigments through vacancy-assisted oxidation pathways. Over extended temporal scales, these radicals continuously react with the red lead pigments through oxidation mechanisms, ultimately resulting in the observed blackened lead dioxide formations seen in historical artifacts.

It is worth noting that the aging reaction of red lead pigments using ROS in an alkaline environment was carried out in a liquid in the study. In the actual environment, the substrate beneath the red lead pigment layer (usually CaCO_3_, CaSO_4_, and Mg_3_[Si_4_O_10_](OH)_2_) also plays a certain role in the aging of the red lead pigment. The aging of red lead occurs at the solid–liquid/gas interface between the mural substrate and the free radicals, under which condition the reaction kinetics are quite slower than in the solution. Further experiments are needed to complete the real-world environment simulation with a long time.

## 4. Conclusions

It has been demonstrated that the reaction of Pb_3_O_4_ with superoxide radicals over varying durations produces the mixtures of partially oxidized Pb_3_O_4_ and PbO_2_ in distinct ratios. The singlet oxygen and superoxide radical made the main contribution to Pb_3_O_4_ oxidation under the experimental conditions. Although the current findings have not yet achieved non-interventive restoration of the original pigment, they provide critical theoretical foundations for interpreting discoloration mechanisms in red lead, which can guide heritage preservation to avoid the high concentration of free radicals in the environment. Future investigations will focus on developing minimally invasive restoration strategies to reverse the oxidation-induced darkening of red lead.

## Data Availability

The original contributions presented in this study are included in the article/Supplementary Materials.

## Supplementary Materials

The following supporting information can be downloaded at https://www.mdpi.com/article/10.3390/molecules30102136/s1, Figure S1: EDS images of the products at different reaction times; Figure S2: Method of measuring whether Pb_3_O_4_ is oxidized in the TMB system by spectrophotometer; Figure S3. (a) The color of the solution after the reaction between TMB and the superoxide-radical-aging products; (b) The UV-Vis absorption spectra of the solution after the reaction between TMB and the superoxide-radical-aging products; Figure S4: The UV-Vis absorption spectra of the solution after the reaction between TMB and the singlet-oxygen-aging products; Table S1: The electronic energy (EE) + thermal free energy correction (TFEC) of each substance calculated under weak alkaline conditions (pH = 8).
