# Supplementary material for: Aging and Discoloration of Red Lead (Pb3O4) Caused by Reactive Oxygen Species Under Alkaline Conditions
Source: Molecules. 2025 May 12;30(10):2136. doi: 10.3390/molecules30102136 (PMC12113696; doi:10.3390/molecules30102136)
Supplement: Supplementary file 1 [file molecules-30-02136-s001.zip › molecules-3577146-supplementary.pdf]

Supplementary Materials

Aging and Discoloration of Red Lead (Pb_3O_4) caused by Reactive Oxygen Species (ROS) under Alkaline Conditions

Zhehan Zhang [1], Qin Huang [1], Jiaxing Sun [1], Qilong Hao [1], Wenyuan Zhang [2], Zongren Yu [2], Bomin Su [2] and Haixia Zhang [1,*]

1 State Key Laboratory of Applied Organic Chemistry, College of Chemistry and Chemical Engineering, Lanzhou University, Lanzhou 730000, China;

2 Gansu Provincial Research Center for Conservation of Dunhuang Cultural Heritage, Dunhuang Academy, Dunhuang 736200, China

* Correspondence: Correspondence: zhanghx@lzu.edu.cn; Tel.: +86-931-8912510

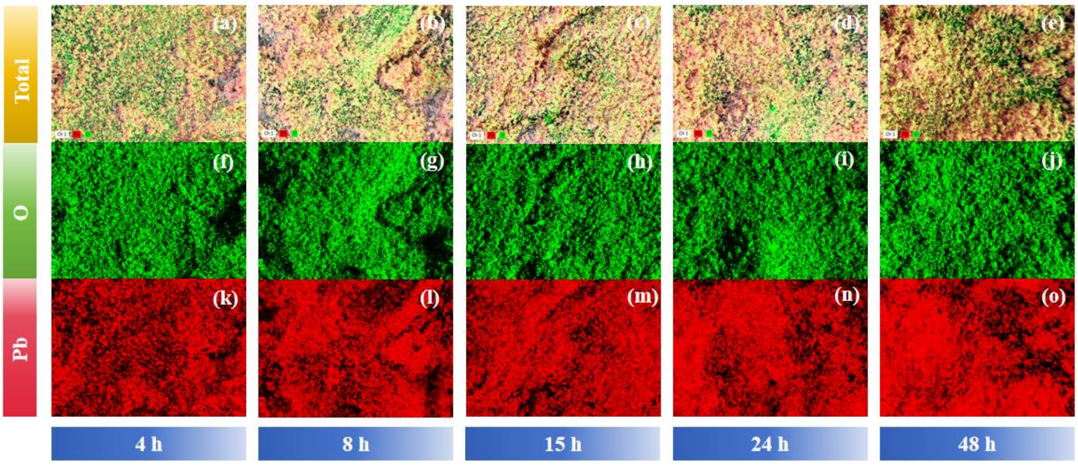

Figure S1. EDS images of (**a**) all the atoms of the 4 h product; (**b**) all the atoms of the 8 h product; (**c**) all the atoms of the 15 h product; (**d**) all the atoms of the 24 h product; (**e**) all the atoms of the 48 h product; (**f**) the O atoms of the 4 h product; (**g**) the O atoms of the 8 h product; (**h**) the O atoms of the 15 h product; (**i**) the O atoms of the 24 h product; (**j**) the O atoms of the 48 h product; (**k**) the Pb atoms of the 4 h product; (**l**) the Pb atoms of the 8 h product; (**m**) the Pb atoms of the 15 h product; (**n**) the Pb atoms of the 24 h product; (**o**) the Pb atoms of the 48 h product.

UV-vis absorption spectra test using TMB system

3,3',5,5'-Tetramethylbenzidine (TMB), a white crystalline powder with odorless and tasteless properties, exhibits poor solubility in water but high solubility in organic solvents such as acetone, diethyl ether, dimethyl sulfoxide (DMSO), and dimethylformamide (DMF). As a novel and safe chromogenic reagent, TMB develops a distinct blue coloration upon oxidation in the system. Pb (IV) demonstrates strong oxidative capability to oxidize TMB, resulting in a blue solution, whereas Pb(II) shows negligible oxidative activity under identical conditions, failing to induce color changes in TMB. This distinct redox behavior enables the detection of Pb (IV) in reaction products using the TMB system coupled with UV-vis spectrophotometry. The steps for detecting whether Pb_3O_4 is oxidized by using the TMB system are shown in Figure S2.

As shown in Figure S3(a), visual observation of solution coloration after TMB addition reveals progressively intensified blue color with increasing reaction time. Corresponding UV-vis absorption spectra (500-800 nm) in Figure S3(b) demonstrate that samples with longer reaction durations exhibit absorption peaks at 650 nm approaching those generated by pure PbO_2 reference solutions. Given the exclusive presence of Pb and O

elements in the analyzed materials, both observations confirm that prolonged reaction times enhance Pb(IV) content in the products, thereby providing additional evidence for the oxidation of Pb(II) to Pb(IV).

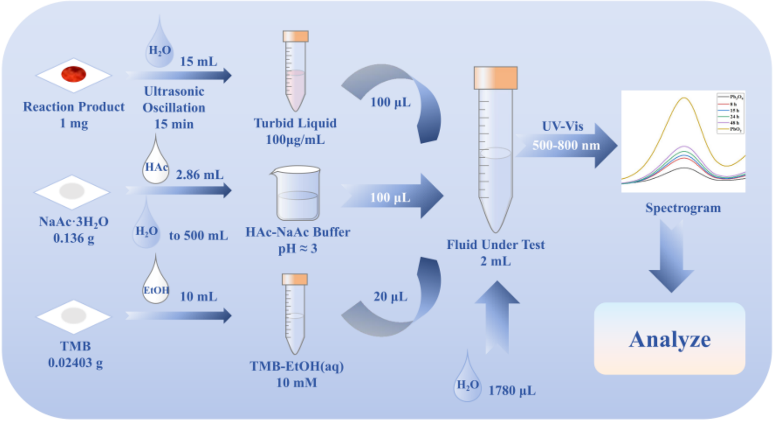

Figure S2. Method of measuring whether Pb_3O_4 is oxidized in TMB system by spectrophotometer.

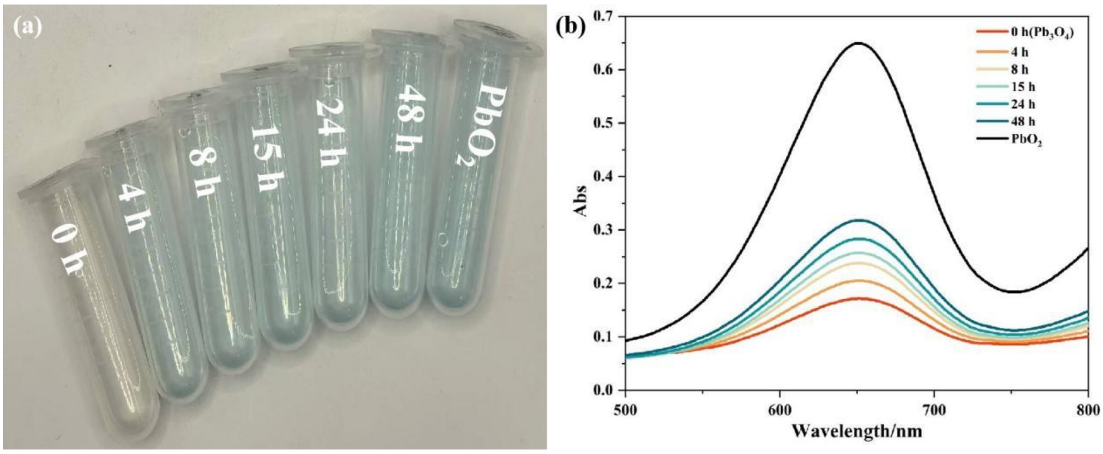

Figure S3. (**a**) The color of the solution after the reaction between TMB and the superoxide-radical-aging products; (**b**) The UV-vis absorption spectra of the solution after the reaction between TMB and the superoxide-radical-aging products.

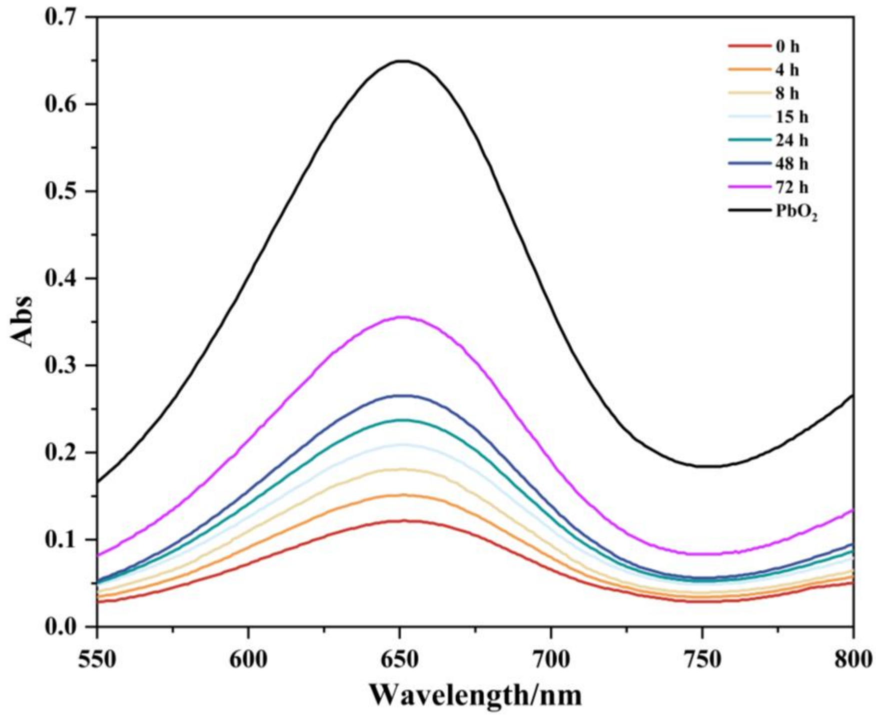

Figure S4. The UV-vis absorption spectra of the solution after the reaction between TMB and the singlet-oxygen-aging products.

Table S1. The Electronic Energy(EE)+Thermal Free Energy Correction(TFEC) of each substance calculated under weak alkaline condition (pH=8).

	**Pb_3O_4**	**PbO_2**	**$^1O_2$**	**$O_2^{-}\cdot$**	**$ONOO^-$**
EE+TFEC/Hartree	-879.27633	-343.254166	-150.28386	-150.46409	-280.42068
	H_2O_2	H_2O	NO_2^-	OH^-	
EE+TFEC/Hartree	-151.601713	-76.432337	-205.25765	-75.86157	
1 Hartree = 2625.5 kJ/mol					
